# Development and validation of a machine learning-based early warning system for predicting venous thromboembolism risk in hospitalized lymphoma patients undergoing chemotherapy: a multicenter and retrospective cohort study

**DOI:** 10.3389/fonc.2025.1566905

**Published:** 2025-08-12

**Authors:** Tingting Jiang, Zailin Yang, Xinyi Tang, Na Fan, Zuhai Hu, Jieping Li, Tingting Liu, Yu Peng, Shuang Chen, Bingling Guo, Xiaomei Zhang, Yong Chen, Jun Li, Dehong Huang, Jun Liu, Yakun Zhang, Xuefen Liu, Xia Wei, Zhanshu Liu, Haike Lei, Yao Liu

**Affiliations:** ^1^ Department of Hematology-Oncology, Chongqing Key Laboratory for the Mechanism and Intervention of Cancer Metastasis, Chongqing University Cancer Hospital, Chongqing, China; ^2^ School of Medicine, Chongqing University, Chongqing, China; ^3^ Department of Medical Administration, Chongqing Public Health Medical Center, Chongqing, China; ^4^ School of Public Health, Chongqing Medical University, Chongqing, China; ^5^ Department of Oncology, The People’s Hospital of Rongchang District, Chongqing, China; ^6^ Department of Hematology, The Third Affiliated Hospital of Chongqing Medical University, Chongqing, China; ^7^ The Affiliated Department of Hematology, Yongchuan Hospital of Chongqing Medical University, Chongqing, China; ^8^ Chongqing Cancer Multi-Omics Big Data Application Engineering Research Center, Chongqing University Cancer Hospital, Chongqing, China

**Keywords:** venous thromboembolism, machine learning, lymphoma, prediction, early warning

## Abstract

**Background:**

Lymphoma patients hospitalized for chemotherapy are at increased risk of venous thromboembolism (VTE) due to prolonged treatment and bed rest. Early prediction of VTE in this group remains challenging. This study aimed to develop a machine learning-based early warning system (VTE-EWS) tailored to these patients.

**Methods:**

Data from 1,141 lymphoma patients hospitalized for chemotherapy were retrospectively collected across four academic medical centers between February 2020 and February 2024. Twelve clinical variables were included, and six machine learning algorithms were applied to build the VTE-EWS. Models were evaluated for accuracy, sensitivity, specificity, and area under the curve (AUC). Variable importance was assessed using permutation analysis, and a nomogram was created to visualize VTE risk. The system’s performance was compared with the Khorana Score (KS).

**Results:**

The training set included 799 patients from Chongqing University Cancer Hospital, with 342 patients from three other centers forming the external validation set. In external validation, all six models demonstrated strong predictive performance, with accuracies ranging from 0.71 to 0.87 and AUCs from 0.78 to 0.84. Six key variables—white blood cell count, D-dimer levels, central venous catheter use, age, chemotherapy cycles, and ECOG performance status—were selected for the nomogram to predict VTE risk visually. Patients with a predicted probability >0.7 were classified as high-risk. The VTE-EWS identified more high-risk patients and provided greater clinical benefit than the KS.

**Conclusions:**

The VTE-EWS leverages simple clinical indicators to quickly and visually predict VTE risk, enabling precise and targeted interventions for lymphoma patients hospitalized undergoing chemotherapy.

## Introduction

Venous thromboembolism (VTE), encompassing deep vein thrombosis (DVT) and pulmonary embolism (PE), is a prevalent cardiovascular disease (1). Factors such as cancer, surgical history, and severe lung disease are all high-risk factors for VTE (2). Notably, cancer patients face a higher risk of VTE in comparison to the general population, with an incidence rate of ~13.9/1000 person-years (3–5). Additionally, VTE serves as a significant contributor to mortality among cancer patients (6, 7). Lymphoma, a malignant hematologic tumor, falls under the category of neoplasms with a high risk of VTE. In a multicenter cohort study that included data from 1995-2012, the incidence of VTE in hospitalized lymphoma patients was as high as 90% (1802490/202289), with a mortality rate as high as 16% (2920/1802490) (6). In recent years, the incidence of VTE has significantly decreased, likely due to the publication of numerous guidelines for VTE management and prevention by academic organizations (8). However, the overall incidence of VTE in lymphoma patients is still approximately 7.9% (9). Among these, diffuse large B cell lymphoma (DLBCL), the most common lymphoma subtype, have a VTE incidence rate as high as 12.8% (10). This may be attributed to the need for long-term chemotherapy and the use of central venous catheters (CVCs) in lymphoma patients, which prolong bed rest and consequently increase the risk of VTE (11, 12). Therefore, precise early warning of the risk of VTE in hospitalized lymphoma patients undergoing chemotherapy is essential.

Currently, the most widely used clinical VTE risk prediction system is the classic Khorana Score (KS) (13, 14). However, it is largely inapplicable to lymphoma patients and is predominantly intended for use in outpatient settings. Given the high heterogeneity of lymphoma and the extended treatment cycles, the KS system is not suitable for hospitalized lymphoma patients undergoing chemotherapy. In 2016, Thorly et al. developed a predictive model for VTE risk in lymphoma patients, providing valuable insights into risk stratification (15, 16). This model fails to specifically account for patients undergoing chemotherapy. It also excludes critical factors, such as the number of chemotherapy cycles and the use of CVCs, both of which significantly impact VTE risk. Additionally, its reliance on extranodal localization, requiring 3–7 days for confirmation, limits its clinical applicability. These limitations highlight the need for a more tailored and accessible predictive system for this high-risk group.

Given these challenges, there is a growing need for new prediction systems. Machine learning algorithms, which can predict patient prognosis more effectively across diverse circumstances, present a promising solution (17). These algorithms offer enhanced predictive capabilities and more powerful processing. However, the complexity of machine learning models can hinder their clinical adoption, primarily due to difficulties in visualization (18). To address this, many models have been integrated with nomograms to improve interpretability and ease of use (19, 20). Therefore, combining machine learning with nomogram-based visualization to create a VTE risk prediction system tailored for hospitalized lymphoma patients undergoing chemotherapy offers a promising approach for widespread clinical adoption.

Herein, we analyzed data from 1,141 lymphoma patients hospitalized for chemotherapy across four medical centers in China to develop and evaluate six machine learning models for VTE risk prediction. To identify the most important variables, we combined variable importance analyses from all models and used a nomogram to assign scores to these key variables. This enabled effective visualization of VTE risk. The early warning system, implemented as an online tool, is designed to rapidly assess VTE risk in hospitalized lymphoma patients undergoing chemotherapy. By improving prediction accuracy, it aims to enhance patient survival and prognosis.

## Methods

### Study design and population

**Figure 1:**
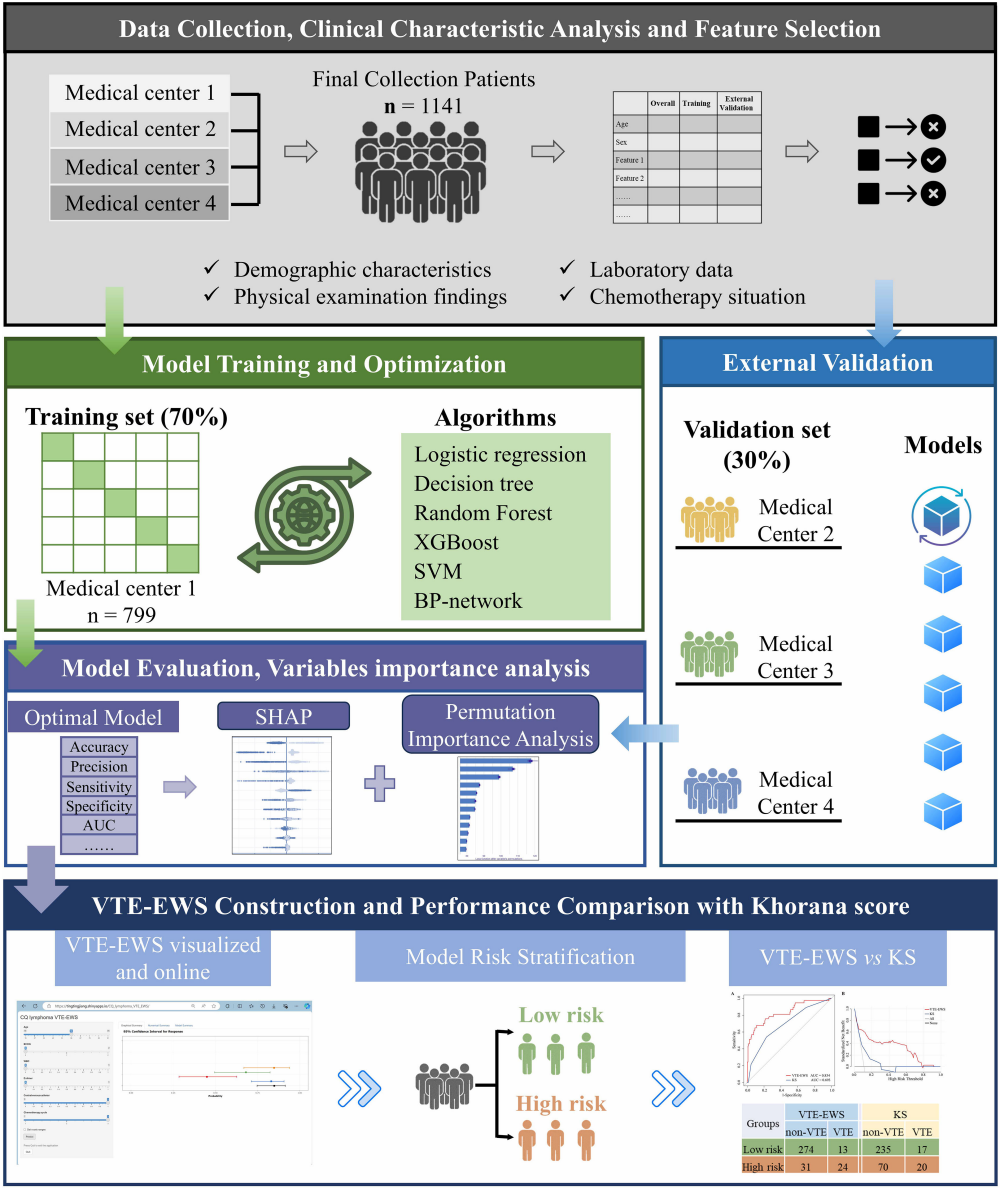
Flow chart of data collection and VTE-EWS construction. SVM, support vector machines; XGBoost, eXtreme Gradient Boosting; BP-network, backpropagation network; KS, khorana score; VTE-EWS, Venous thromboembolism-early warning system.

In this study, a total of 1141 patients who hospitalized to receive chemotherapy in four academic medical centers, as Chongqing University Cancer Hospital (CQUCH), Yongchuan Hospital of Chongqing Medical University (YCHCQMU), Third Affiliated Hospital of Chongqing Medical University (TAHCQMU), and People’s Hospital of Rongchang District (PHRC), from February 2020 to February 2024 were retrospectively analyzed (**Figure 1**). This retrospective cohort study aimed to assess the risk of VTE in hospitalized lymphoma patients undergoing chemotherapy. According to the Chinese Society of Clinical Oncology guidelines for the diagnosis and treatment of lymphoma, patients with a confirmed lymphoma diagnosis can be hospitalized regularly to receive chemotherapy based on their specific disease conditions.

The Ethics Committee of Chongqing University Cancer Hospital granted the necessary ethical approval. Inclusion criteria for this study encompassed: patients >18 years; a histopathological diagnosis of lymphoma; those who had hospitalized for chemotherapy at least once. The exclusion criteria were: patients with unknown VTE status; those with unknown histological type; patients with unknown Ann Arbor stage; patients with unknown treatment; and patients with unknown other required information. Subsequent screening utilized the detailed inclusion and exclusion criteria. This study was in line with the guidelines of the Declaration of Helsinki.

### Chemotherapy regiments

Treatment options vary depending on the subtype of lymphoma. Hodgkin lymphoma is primarily treated with the classical ABVD protocol, which includes doxorubicin, bleomycin, vinblastine, and dacarbazine. B cell lymphoma is commonly treated with CHOP (cyclophosphamide, doxorubicin, vincristine, and prednisone) or EPOCH (etoposide, doxorubicin, vincristine, cyclophosphamide, and prednisone), often in combination with rituximab. T cell lymphoma is primarily treated with CHOP or EPOCH, or with the Hyper-CVAD regimen (cyclophosphamide, doxorubicin, vincristine, and prednisone) combined with high-dose methotrexate and cytarabine. For NK/T cell lymphoma, the DDGP protocol, consisting of dexamethasone, cisplatin, gemcitabine, and pegaspargase, is recommended. The selection of these regimens is tailored to the patient’s lymphoma subtype, disease stage, and overall systemic condition.

### Study outcomes and VTE diagnosis

The primary outcome was the occurrence of VTE among lymphoma patients undergoing chemotherapy in this study. Before admitting lymphoma patients for a new cycle of chemotherapy, clinicians need to assess their physical condition, which includes screening for VTE. VTE diagnostic procedures took place during the patients’ chemotherapy hospitalization. DVT diagnosis relied on either Doppler ultrasound or venography. In contrast, PE was diagnosed through CT pulmonary arteriography (CTPA) or nuclear lung ventilation/perfusion imaging (21). Thrombosis was defined by the presence of incompressible venous segments, observable thrombus formation, and the detection of residual flow in veins exhibiting vascular filling defects through Doppler. The study reported no false positive cases based on imaging.

### Features selection

Features chosen for the machine learning models were derived from routinely collected data of lymphoma patients. The lasso regularization analysis was used to screen features with the clinical information of lymphoma patients in the training set, excluding those with a coefficient of zero. Additionally, variables with high collinearity, excessive missing values, or limited clinical interpretability were deprioritized. In conclusion, a total of 12 features were retained as variables based on the screening results and clinical expertise. These variables encompassed: age, sex, body mass index (BMI), CVCs use, Eastern Cooperative Oncology Group performance status (ECOG score), histological types, Ann Arbor stage, white blood cell (WBC) count, hemoglobin (HB) level, D-dimer level, platelet count (PLT), and the number of chemotherapy cycles. The BMI scoring criteria are derived from the WHO, and the calculation formula is weight (kg)/height (m)². Finally, the correlation of the selected 12 variables was analyzed using the “corrplot” package. For VTE patients, data were collected during the most recent hospitalization prior to the VTE event. For non-VTE patients, data were collected during their last hospitalization within the study inclusion period.

### Model development

The dataset was primarily divided into a training set (70%, n = 799) and an independent validation set (30%, n = 342) (**Figure 1**). The training set data was primarily sourced from CQUCH, while the validation set data was mainly obtained from three external medical centers: YCHCQMU, TAHCQMU, and PHRC. Statistical tests revealed no significant differences between the two cohorts (*p* > 0.05). Given the low incidence of VTE in lymphoma patients, class imbalances in our data are inevitable. To rectify this, resampling techniques like the ROSE and SMOTE algorithms are utilized to balance the training dataset. We evaluated three resampling methods (undersampling, oversampling, and mixed sampling), where mixed sampling had the best effect. Subsequently, six machine learning models were developed to predict the risk of VTE (1): logistic regression; (2) random forest; (3) backpropagation network (BP-network); (4) XGBoost; (5) decision tree; and (6) SVM (**Figure 1**). The hyperparameter tuning was conducted for all models using a 10-fold cross-validation method. The final models were then built using the optimal hyperparameters along with the balanced training set.

### Model evaluation

The predictive performance of the models was ascertained by comparing and analyzing the accuracy, precision, sensitivity, specificity, F1 score, brier score, and area under curve (AUC) for both the training and validation sets. The Brier score measures the gap between the predicted probability of an outcome and the true outcome, with a lower Brier score indicating better model performance. The receiver operator characteristic (ROC) curves were derived from the rate of true positives (sensitivity) against the rate of false positives (1-specificity). The AUC and ROC curves demonstrated the model’s ability to differentiate between outcomes. To finalize the evaluation of the top-performing model, calibration curves and decision curves were used. Calibration curves gauge how closely a classification model’s predicted probability aligns with the actual probability, while decision curve analysis (DCA) assigns varying weights to different misclassification types, offering direct clinical benefit (22).


$$Accuracy=\frac{True\;positive+True\;negative}{True\;positive+True\;negative+False\;positive+False\;negative}$$

$$Precision=\frac{True\;positive}{True\;positive+False\;positive}$$

$$F1\;score=\frac{2\;\times \;Precision\;\times \;Sensitivity}{Precision+Sensitivity}$$

$$Sensitivity=\frac{True\;positive}{True\;positive+False\;negative}$$

$$Specificity=\frac{True\;negative}{True\;negative+False\;positive}$$

Brier score=∑(pi−oi)/N

### Variables importance analysis

We investigate the variables importance for the XGBoost model using SHapley Additive exPlanations (SHAP) values. This was achieved through the “SHAPforxgboost” package, and the findings were visualized using the “ggplot2” package. To comprehensively evaluate variable importance, we employed the “DALEX” package to create model explainers for each model and used permutation importance analysis to compute loss functions and obtain variable importance rankings. Permutation importance analysis provides an objective measure of how much each variable contributes to the overall model performance. This is achieved by randomly rearranging each variable and observing the consequent changes in model performance. Although this method does not yield p-values like traditional statistical testing, it is widely recognized in machine learning as a robust, model-agnostic approach to assess predictor relevance—especially suitable for nonlinear models. Subsequently, the results of permutation importance analysis for each model were integrated and standardized. We thoroughly assessed the overall ranking of variables’ importance by creating a heatmap.

### VTE-EWS visualization and application

Model visualization is a crucial task in machine learning. We used a nomogram as a visualization tool to demonstrate the model’s predictive ability and the contribution of each variable to the outcomes (23). Based on the results of the permutation importance analysis, we selected the top six variables from the overall ranking and employed the “rms” package to construct the nomogram. Finally, we utilized the “DynNom” package to develop an online prediction tool based on the nomogram.

### Comparison of the VTE-EWS with KS

Based on the *VTE-EWS* and the classic KS, we first identified the high-risk and low-risk VTE groups. Accuracy, precision, sensitivity, specificity, and AUC were used to evaluate the discriminative ability of the model in both the training and the external validation sets. Additionally, we assessed the model’s performance and clinical benefit using ROC curves and DCA.

### Data analysis

Missing data were imputed using multiple interpolations from the mice package. All subsequent analyses were conducted using the imputed data. Continuous variables with a non-normal distribution were analyzed using the Mann-Whitney U test and are expressed as median (interquartile range). Categorical variables were analyzed with the chi-square test or Fisher’s exact test and are expressed as count (percentage). The KS had identified five variables, and patients scoring 3 or higher were classified as high-risk VTE group (13). All models and statistical evaluations were conducted using RStudio (version 2023.06.2-551) and R (version 4.3.3).

## Results

### Characteristics of subjects

From 2020 to 2024, our study screened a total of 1337 individuals diagnosed with lymphoma. As a result, 1,141 patients (799 patients in the training set, 342 patients in the external validation set) were considered for the analysis (**Table 1**; **Supplementary Table S1**–**S3**). The ratio of VTE in the training set and the external validation set were 10.89% (n = 87) and 10.82% (n = 37), respectively, indicating very similar rates. The ratio of male-to-female lymphoma patients in totally was approximately 7:5, with a median age of 56.00 [48.00, 66.00] years. The median age of patients with VTE was 64.5 years, while for patients with non-VTE, it was 56 years (**Supplementary Table S2**). Based on the histological types, patients were divided into four categories: Hodgkin lymphoma (109 cases, 9.6%), B cell lymphoma (839 cases, 73.5%), T cell lymphoma (97 cases, 8.5%), and NK/T cell lymphoma (96 cases, 8.4%). The distribution of these histological types in both the training set and the validation set was consistent with their overall distribution. Approximately 65% of the patients were classified under Ann Arbor Stage III/IV. In the training set and validation set, 46% (40/87) and 43.2% (16/37) of VTE patients, respectively, used CVCs. In contrast, the CVCs’ usage rate among non-VTE patients in both groups were only between 4% and 7%. Most patients (>90%) underwent 1 to 5 cycles of chemotherapy, but the proportion of VTE patients significantly increased (>16%) among those undergoing 6 to 10 cycles. Additional routine indicators are presented in **Table 1**.

**Table 1:** Clinical demographics and clinicopathologic characteristics of hospitalized lymphoma patients undergoing chemotherapy.

Variables	Overall N=1141	Training set		*p*	Validation set		*p*
		non-VTE N=712	VTE N=87		non-VTE N=305	VTE N=37	
Age, (median [IQR])	56.00 [48.00, 66.00]	56.00 [47.00;66.00]	64.00 [55.00;70.00]	<0.001	55.00 [47.00;65.00]	66.00 [53.00;68.00]	0.023
Sex, No. (%)				0.018			0.303
Female	678 (59.40)	409 (57.40)	62 (71.30)		188 (61.60)	19 (51.40)	
Male	463 (40.60)	303 (42.60)	25 (28.70)		117 (38.40)	18 (48.60)	
BMI, No. (%)				0.501			0.294
< 24	687 (60.20)	425 (59.70)	50 (57.50)		193 (63.30)	19 (51.40)	
24-28	375 (32.90)	238 (33.40)	28 (32.30)		93 (30.50)	16 (43.20)	
≥ 28	79 (6.90)	49 (6.88)	9 (10.30)		19 (6.23)	2 (5.41)	
CVCs, No. (%)				<0.001			<0.001
No	1021 (89.50)	661 (92.80)	47 (54.00)		292 (95.70)	21 (56.80)	
Yes	120 (10.50)	51 (7.16)	40 (46.00)		13 (4.26)	16 (43.20)	
ECOG, No. (%)				0.288			0.794
0 point	356 (31.20)	219 (30.80)	27 (31.00)		97 (31.80)	13 (35.10)	
1 point	598 (52.40)	382 (53.70)	41 (47.10)		158 (51.80)	17 (45.90)	
> 2 points	187 (16.40)	111 (15.60)	19 (21.80)		50 (16.40)	7 (18.90)	
Histological type, No. (%)				0.091			0.289
Hodgkin	109 (9.60)	69 (9.69)	6 (6.90)		30 (9.84)	4 (10.80)	
B cell	839 (73.50)	524 (73.60)	59 (67.80)		232 (76.10)	24 (64.90)	
T cell	97 (8.50)	58 (8.15)	14 (16.10)		20 (6.56)	5 (13.50)	
NK/T cell	96 (8.40)	61 (8.57)	8 (9.20)		23 (7.54)	4 (10.80)	
Ann Arbor, No. (%)				0.216			0.985
I-II	398 (34.90)	257 (36.10)	25 (28.70)		104 (34.10)	12 (32.40)	
III-IV	743 (65.10)	455 (63.90)	62 (71.30)		201 (65.90)	25 (67.60)	
WBC, No. (%)				<0.001			<0.001
< 11 ×10 ^9^/L	976 (85.50)	653 (91.70)	35 (40.20)		273 (89.50)	15 (40.50)	
≥ 11 ×10 ^9^/L	165 (14.50)	59 (8.29)	52 (59.80)		32 (10.50)	22 (59.50)	
HB, No. (%)				0.001			0.689
≥ 100 g/L	921 (80.70)	583 (82.00)	58 (66.70)		32 (10.50)	21 (56.80)	
< 100 g/L	220 (19.30)	128 (18.00)	29 (33.30)		273 (89.50)	16 (43.20)	
PLT, No. (%)				0.177			0.803
< 350 ×10 ^9^/L	1033 (90.50)	650 (51.30)	75 (19.50)		275 (90.20)	33 (89.20)	
≥ 350 ×10 ^9^/L	108 (9.50)	62 (8.71)	12 (13.80)		30 (9.84)	4 (10.80)	
D-dimer, No. (%)				<0.001			0.018
≤ 0.5 mg/L	542 (47.50)	365 (51.30)	17 (19.50)		150 (49.20)	10 (27.00)	
> 0.5 mg/L	599 (52.50)	347 (48.70)	70 (80.50)		155 (50.80)	27 (73.00)	
Chemotherapy cycles, No. (%)				<0.001			<0.001
1-5	1049 (91.90)	669 (94.00)	69 (79.30)		285 (93.40)	26 (70.30)	
6-10	69 (6.00)	31 (4.35)	14 (16.10)		14 (4.59)	10 (27.00)	
≥ 11	23 (2.00)	12 (1.69)	4 (4.60)		6 (1.97)	1 (2.70%)	

BMI, body mass index; CVCs, central venous catheters; ECOG, Eastern Cooperative Oncology Group performance status; IQR, interquartile range; WBC, white blood cell count; HB, hemoglobin; PLT, Platelet count; VTE, venous thromboembolism.

Twelve variables were ultimately included in the analysis. All selected variables had less than 10% missing data (**Supplementary Table S3**). A correlation heatmap (**Supplementary Figure S1**) revealed weak interactions between the variables, with Pearson correlation coefficients below |0.4|. This indicates that the variables can be included in the analysis simultaneously without affecting the subsequent results.

### Model performance evaluation and variable importance analysis

**Figure 2:**
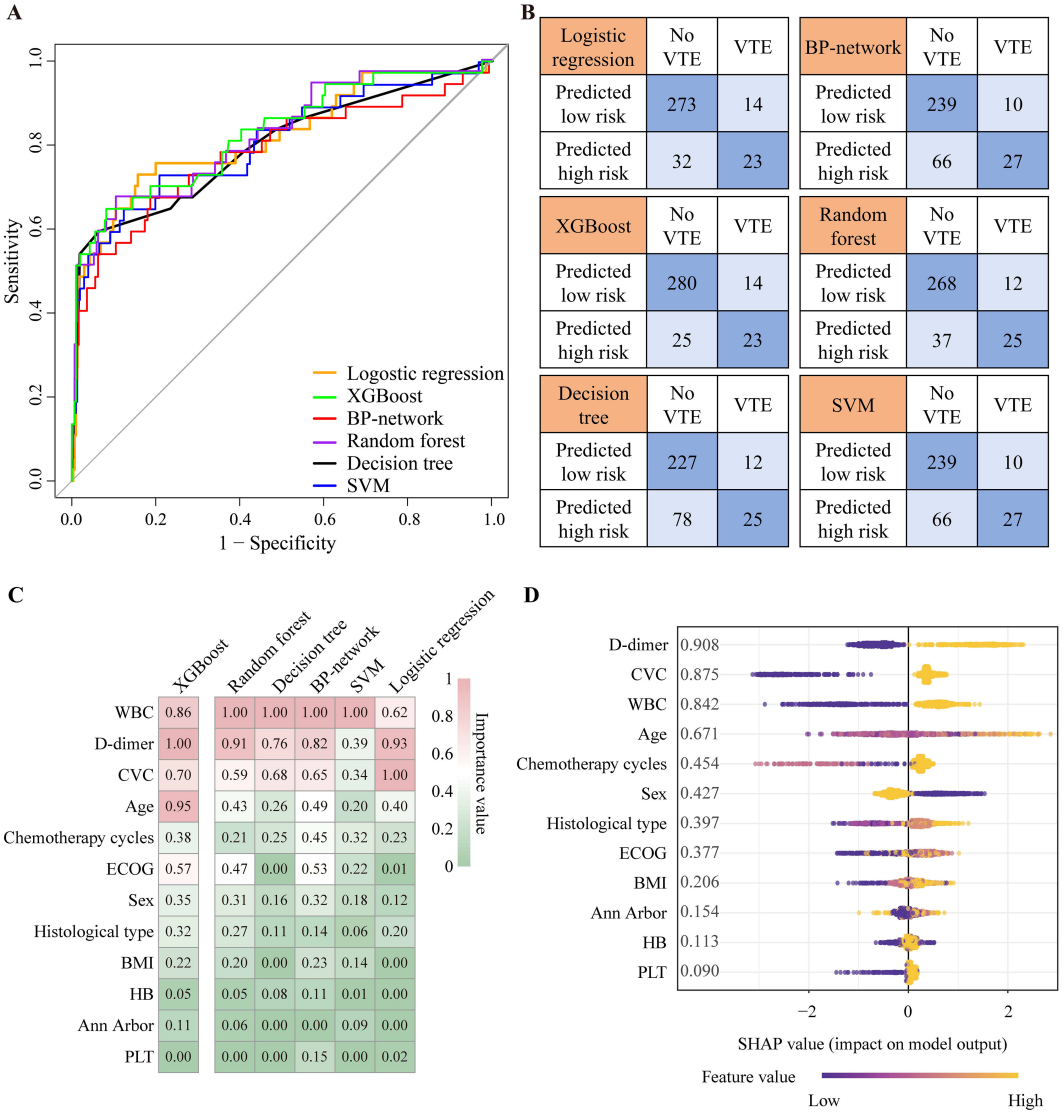
The ROC curve and confusion matrix of the six machine learning models in validation set. **(A)** ROC curve of validation set. **(B)** Confusion matrix of validation set. **(C)** The heatmap of variable importance analysis for models by permutation importance analysis. **(D)** SHAP analysis for XGBoost model. XGBoost, eXtreme Gradient Boosting; SVM, support vector machines; BP-network, backpropagation network; VTE, venous thromboembolism; ROC curve, receiver operator characteristic curve; BMI, body mass index; CVC, central venous catheter; ECOG, Eastern Cooperative Oncology Group performance status; WBC, white blood cell count; HB, hemoglobin; PLT, Platelet count; SHAP, SHapley Additive exPlanations; XGBoost, eXtreme Gradient Boosting.

Out of the 1,141 patients included in the analysis, 124 developed VTE during their hospitalization for chemotherapy, all of which occurred after chemotherapy initiation. ROC curves and confusion matrix assessed the discriminatory capability of the six models (**Figure 2**). In the external validation set, the ROC curves of all models are similar, indicating that their differentiation ability is comparable (**Figure 2A**). The similar results also appeared in the training set (**Supplementary Figure S2**). In the confusion matrix, all models predicted between 23 and 27 VTE patients and between 239 and 280 non-VTE patients. Among them, XGBoost predicted the most non-VTE patients (**Figure 2B**). Utilizing the ROC curves and the data from the confusion matrix, we compared the accuracy, precision, sensitivity, specificity, F1 score, brier score, and AUC across the six models (**Supplementary Table S4**). In the external validation set, the performance of all models was very similar, with accuracies ranging from 0.74 to 0.89, AUCs from 0.80 to 0.83, sensitivities from 0.62 to 0.73, specificities from 0.74 to 0.92, F1 scores from 0.36 to 0.50, and brier scores from 0.14 to 0.40. XGBoost had the highest accuracy (0.89) and the lowest Brier score (0.14), suggesting that it possesses a reduced rate of misdiagnosis and superior predictive reliability. The calibration curves of XGBoost demonstrated excellent predictive accuracy, and DCA indicated substantial clinical net benefit (**Supplementary Figure S3**).

Subsequently, we examined the variable importance for all models by permutation importance analysis and presented the findings in a heatmap for comparison. The heatmap revealed that WBC, D-dimer, CVCs, age, chemotherapy cycles, and ECOG score were the top six influential variables (**Figure 2C**; **Supplementary Table S5**). This overall ranking order aligns with the results of the permutation importance analysis in SHAP of XGBoost (**Supplementary Table S5**; **Figure 2D**). SHAP analysis reveals how various levels of these variables impact the occurrence of VTE. As shown in **Figure 2D**, factors such as a WBC count of ≥ 11 × 10^9^/L, D-dimer levels > 0.5 mg/L, the use of CVC, advanced age, an increased number of chemotherapy cycles, and higher ECOG scores are all associated with an increased risk of VTE.

### VTE-EWS visualization and application

**Figure 3:**
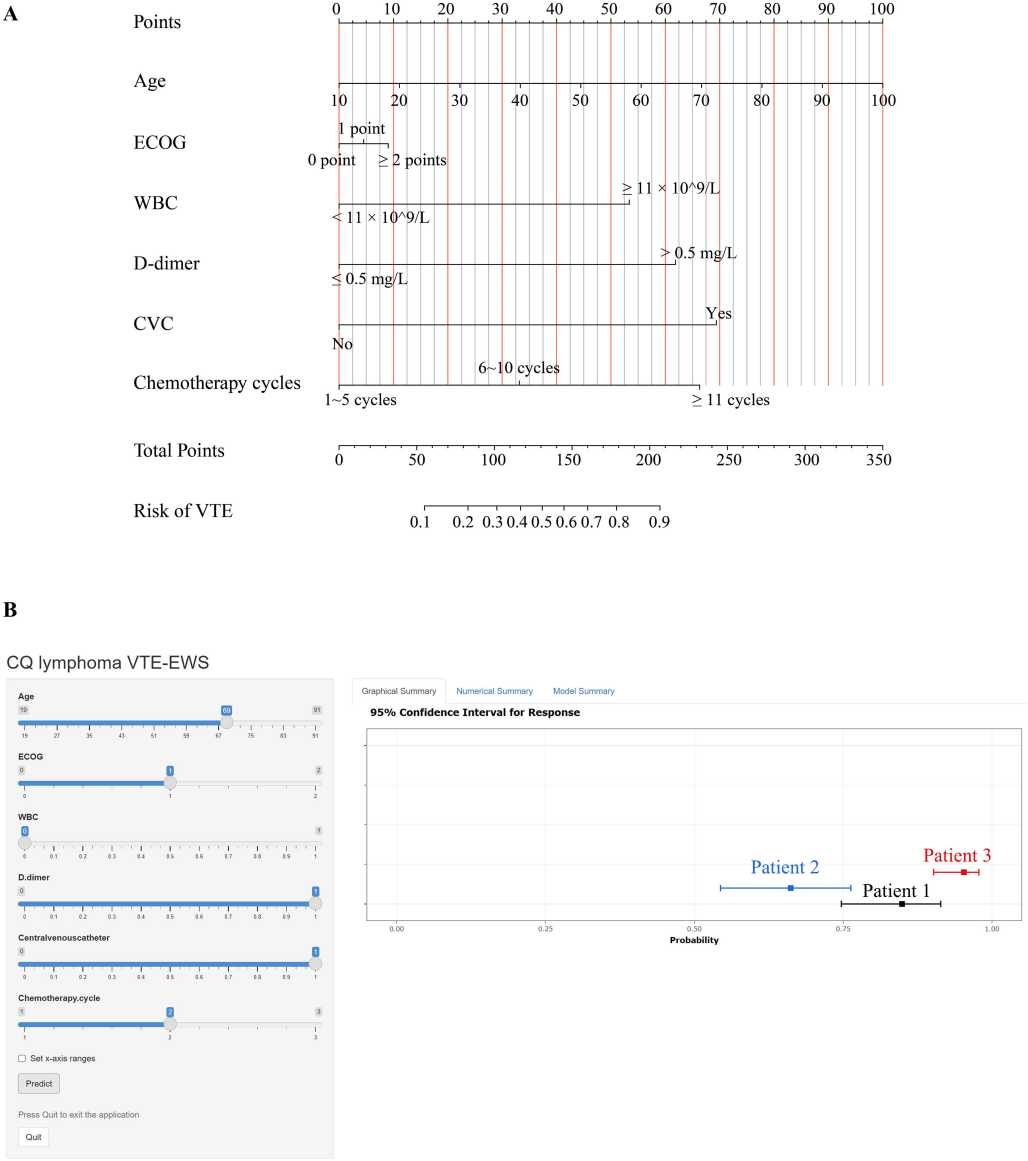
The construction of VTE-EWS. **(A)** A nomogram to predict visually the risk of VTE in hospitalized lymphoma patients undergoing chemotherapy. **(B)** The interface of CQ lymphoma VTE-EWS. Users can fill in the corresponding item and press the “Predict” button to obtain a VTE risk profile. CVC, central venous catheter; ECOG, Eastern Cooperative Oncology Group performance status; WBC, white blood cell count; VTE, venous thromboembolism.

Based on the comprehensive analysis of variable importance, we selected six key variables: WBC, D-dimer, CVC, age, chemotherapy cycles, and ECOG score. These six variables were selected for the final nomogram model due to the stability and agreement of these variables across all models, which enhances robustness, and because each predictor has strong clinical associations with VTE, supporting both model interpretability and clinical applicability. We utilized a nomogram to assign scores to these variables, thereby visualizing the model’s prediction results. Each clinical parameter was graphically assigned a score, with the corresponding vertical line labeled as the point axis. The total score is then located on the total score axis to determine the probability of VTE risk for hospitalized lymphoma patients undergoing chemotherapy (**Figure 3A**).

Finally, we transformed the nomogram into a web-based VTE risk prediction tool, creating a comprehensive and convenient early warning system (**Figure 3B**) (https://tingtingjiang.shinyapps.io/CQ_lymphoma_VTE_EWS/). We named this system CQ lymphoma VTE-EWS (hereinafter referred to as “VTE-EWS”). Clinicians only need to input patient characteristics on the webpage and click the “Predict” button below. The tool then processes these inputs through VTE-EWS and displays the patient’s VTE risk result on the right-hand side. Patients with a predicted risk probability exceeding 0.7 are classified as high-risk for VTE.

### Comparison of the visualized VTE-EWS with KS

**Figure 4:**
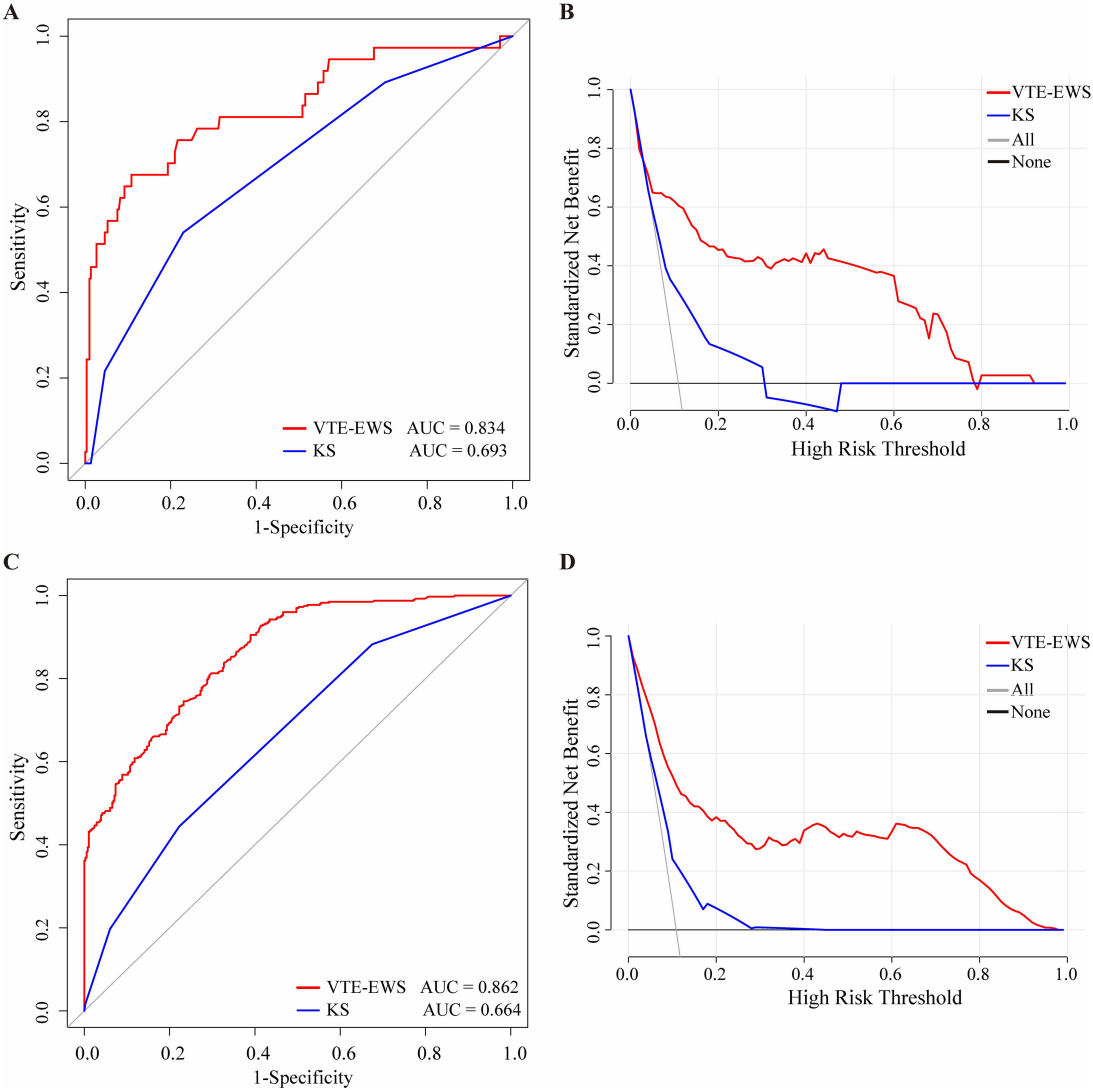
The ROC curve and DCA comparison of the VTE-EWS and KS in the training set and the external validation set. **(A)** The ROC curve comparison of the VTE-EWS and KS in the external validation set. **(B)** The DCA comparison of the VTE-EWS and KS in the external validation set. **(C)** The ROC curve comparison of the VTE-EWS and KS in the training set. **(D)** The DCA comparison of the VTE-EWS and KS in the training set. AUC, area under curve; KS, Khorana score; VTE-EWS, venous thromboembolism-early warning system.

The results clearly exhibited that in the external validation set, the VTE-EWS achieved a non-VTE detection rate of 90% (277/305, specificity = 0.91, 95%*CI*: 0.87-0.94) among the low-risk group, compared to 77% (235/305, specificity = 0.77, 95%*CI*: 0.72-0.81) for the KS (**Supplementary Tables S6**, **S7**). For the high-risk group in the external validation set, the VTE-EWS had a VTE detection rate of 65% (24/37, sensitivity = 0.65, 95%*CI*: 0.49-0.78), while the KS had a detection rate of 54% (20/37, sensitivity = 0.54, 95%*CI*: 0.38-0.69) (**Supplementary Tables S6**, **S7**). In the training set, the performance of the VTE-EWS was also significantly higher than that of the KS (**Supplementary Table S7**). The ROC curves indicated that the VTE-EWS demonstrated a higher AUC in both the training set (VTE-EWS:0.86, 95%*CI*:0.84-0.87, KS: 0.66, 95%*CI*: 0.63- 0.70) and the external validation set (VTE-EWS: 0.83, 95%*CI*: 0.75-0.91; KS: 0.69, 95%*CI*: 0.61-0.78) (**Figure 4A, C**). Similarly, the DCA showed that the VTE-EWS provided greater clinical net benefit than the KS in both the training set (VTE-EWS: 1%~98%, KS: 5%~28%) and the external validation set (VTE-EWS: 1%~78%, KS: 5%~30%) (**Figures 4B, D**).

By observing the distribution of patients for each KS score, we found that most VTE patients had scores ranging from 2 to 4 (**Supplementary Figure S4**). However, the classical KS considers patients with a score of 3 or higher to be high-risk for VTE, which is one of the reasons why this model is not applicable to hospitalized lymphoma patients undergoing chemotherapy.

## Discussion

Hospitalized lymphoma patients have a high prevalence of VTE and an elevated mortality rate (6). As a result, the prevention and treatment of VTE have become a vital part of comprehensive cancer care (24). In this study, we included variables such as the number of chemotherapy cycles and the utilization of CVC, which are strongly associated with VTE risk in hospitalized lymphoma patients undergoing chemotherapy, addressing the limitations of existing models. The objective was to build a high-performance machine-learning predictive model to assess VTE risk factors in hospitalized lymphoma patients undergoing chemotherapy and to provide visualized explanations for clinical VTE prevention. This prediction model can potentially improve patient outcomes by providing early warnings of VTE incidence.

In this study, we utilized routine indicators from hospitalized lymphoma patients as variables. These variables originate from three sources: patient-related factors, tumor-related factors, and laboratory biomarkers, all of which are more convenient for clinical application. Six machine learning models (logistic regression, random forest, BP-network, XGBoost, decision tree, SVM) were examined in this study. All models exhibited strong predictive performance.

One of the crucial tasks in constructing a machine learning model is selecting variables that significantly influence the predicted outcome. Therefore, we combined the results of the variable importance analyses from all six models to provide a comprehensive assessment of each variable’s importance. This approach rationalizes the selection of variables that significantly impact the risk of VTE occurrence. Based on this methodology, we identified the top six influential factors as WBC, D-dimer, CVCs’ use, age, chemotherapy cycles, and ECOG score. SHAP analysis has aided in understanding how changes in variable levels influence the risk of VTE.

WBC are pivotal in immunity, yet their role in cancer-associated VTE remains unclear. Tumors release inflammatory cytokines, activating tissue factor on monocytes and promoting fibrin deposition (25–27). Khorana et al. reported a two-fold increased VTE risk in cancer patients with leukocytosis prior to chemotherapy and a 3% incidence in those with persistent leukocytosis after one cycle (13, 28–30). Our analysis confirmed elevated WBCs as a significant risk factor. Similarly, D-dimer, a fibrin degradation product, strongly correlates with VTE, particularly post-surgery (31–33). We observed that sustained D-dimer elevations were critical in predicting VTE risk, reinforcing its clinical utility.

CVCs, essential for chemotherapy, notably increase VTE risk due to vascular injury and related factors (34–36). Prolonged chemotherapy further elevates VTE risk through increased coagulation activity and inflammatory markers (37). Advanced age, another key factor, correlates with VTE in cancer patients, including lymphoma (38–41). Poor functional status, reflected by ECOG scores ≥ 2, also predicts VTE, as noted by Michela et al. (42–44). Our findings identified age and ECOG score as significant predictors of VTE, emphasizing the importance of preventive measures for hospitalized lymphoma patients, especially those with prolonged bed rest, CVCs’ use, or undergoing multiple chemotherapy cycles, all of which further elevate VTE risk.

The ultimate goal of developing the machine learning model is to utilize the selected key indicators to construct a VTE early warning system and achieve its visualization. Previous machine learning models often lack visualization and interpretability (18, 45). Although the nomogram model is visual and interpretable, it has limitations in analyzing complex data due to its reliance on basic statistical methods. Therefore, we combined the advantages of both approaches to develop an online nomogram tool based on machine learning models. In this study, we assigned scores to the variables based on the comprehensive assessment of their importance and used a nomogram to present the predictive weights of each variable in the form of scores (23). This method effectively visualizes the model’s predictive efficacy and highlights the impact of key variables on the target event.

Moreover, compared to the classical KS, our lymphoma-specific VTE-EWS accurately identifies high-risk VTE patients with a lower misdiagnosis rate, and demonstrates superior predictive performance and clinical net benefit. In 2016, Thorly developed a predictive model for VTE in lymphoma patients, but it did not address predictors specific to hospitalized patients undergoing chemotherapy (15, 16). Key factors, such as the number of chemotherapy cycles and the use of CVCs, which significantly increase VTE risk due to prolonged bed rest, were excluded from the analysis. Moreover, the model required extranodal localization data, which typically takes 3–7 days to obtain, potentially delaying clinical decision-making. As a result, its clinical utility is limited.

In contrast, our VTE-EWS offers a rapid, online, and visually intuitive tool for predicting VTE risk. It enables clinicians to quickly assess the risk for hospitalized lymphoma patients undergoing chemotherapy using desktops or mobile devices. By incorporating routinely collected variables before each chemotherapy, such as age, ECOG, WBC count, D-dimer, the number of chemotherapy cycles and CVCs use, the model supports real-time risk stratification during inpatient care. Patients with a predicted risk probability exceeding 0.7 are classified as high-risk, allowing for timely interventions that support early prevention and improved outcomes.

## Limitations

Several limitations were shown in this study. Firstly, our study focused on hospitalized lymphoma patients and may not be applicable to outpatients. Secondly, since the study was retrospective, this VTE early warning system has not been validated with prospective data. Thirdly, although external validation was performed using an independent dataset, all study sites were located in the same province of China. In the next phase, we will undertake a prospective study to implement and optimize the model across different provinces to enhance its generalizability.

## Conclusions

In summary, rather than focusing solely on constructing machine learning models, we evaluated multiple models and identified routine clinical variables—WBC, D-dimer, CVC use, age, chemotherapy cycles, and ECOG score—that effectively provide early warnings of VTE. These variables were incorporated into an online visualization tool, creating the VTE-EWS. Built using multicenter data, this system minimizes bias and enhances reliability. Patients with a predicted risk probability above 0.7 are classified as high-risk for VTE, enabling timely and targeted interventions. We recommend that clinicians assess VTE risk before each hospitalization for chemotherapy, considering admission-specific indicators. In the future, we will develop an intelligent prediction system based on the electronic medical record system.

## Data Availability

The raw data supporting the conclusions of this article will be made available by the authors, without undue reservation.
