# Supplementary material for: Development and validation of a machine learning-based early warning system for predicting venous thromboembolism risk in hospitalized lymphoma patients undergoing chemotherapy: a multicenter and retrospective cohort study
Source: Front Oncol. 2025 Aug 12;15:1566905. doi: 10.3389/fonc.2025.1566905 (PMC12378646; doi:10.3389/fonc.2025.1566905)
Supplement: Supplementary file 1 [file DataSheet1.docx]

***Supplementary Material***

**Development and Validation of a Machine Learning-Based Early Warning System for Predicting Venous Thromboembolism Risk in Hospitalized Lymphoma Patients Undergoing Chemotherapy: a multicentre and retrospective cohort study**

Tingting Jiang ^a, #^, Zailin Yang ^a, #^, Xinyi Tang ^a, c, #^, Na Fan ^d, #^, Zuhai Hu ^e^, Jieping Li ^a^, Tingting Liu ^a^, Yu Peng ^a^, Shuang Chen ^a^, Bingling Guo ^a^, Xiaomei Zhang ^a^, Yong Chen ^h^, Jun Li ^a^, Dehong Huang ^a^, Jun Liu ^a^, Yakun Zhang ^a,c^, Xuefen Liu ^h,^*, Xia Wei ^g,^*, Zhanshu Liu ^f,^*, Haike Lei ^b,^*, Yao Liu ^a,^*

^a^ Department of Hematology-Oncology, Chongqing Key Laboratory of Translational Research for Cancer Metastasis and Individualized Treatment, Chongqing University Cancer Hospital, Chongqing, 400030, China

^b^ Chongqing Cancer Multi-omics Big Data Application Engineering Research Center, Chongqing University Cancer Hospital, Chongqing, 400030, China

^c^ School of Medicine, Chongqing University, Chongqing, 400030, China

^d^ Chonging Public Health Medical Center, Chongqing, 400036, China

^e^ School of Public Health, Chongqing Medical University, Chongqing, 401331, China

^f^ The affiliated Department of Hematology, Yongchuan Hospital of Chongqing Medical University, Chongqing 402160, China.

^g^ Department of Hematology, The Third Affiliated Hospital of Chongqing Medical University, Chongqing, 400016, China

^h^ The People’s Hospital of Rongchang District, Chongqing, 402400, China

^#^ These authors contributed equally to this work.

^*^ Corresponding authors: Yao Liu: liuyao77@cqu.edu.cn, HaiKe Lei: [tohaike@163.com](mailto:tohaike@163.com), Zahnshu Liu: [liuzhanshu@163.com](mailto:liuzhanshu@163.com), Xia Wei: [10988992@qq.com](mailto:10988992@qq.com), Xuefen Liu: lxfpd@sina.com

**Table S1.** Clinical demographics and clinicopathologic characteristics of in training set and validation set

| **Variables** | **Train cohort**  N=799 | **Validation cohort**  N=342 | ***p*** |
| --- | --- | --- | --- |
| Age, (median [IQR]) | 56.0 [47.5; 66.5] | 55.0 [48.0; 66.0] | 0.490 |
| Sex, No. (%) |  |  | 0.666 |
| Female | 471 (58.9%) | 207 (60.5%) |  |
| Male | 328 (41.1%) | 135 (39.5%) |  |
| BMI, No. (%) |  |  | 0.658 |
| 24 | 475 (59.4%) | 212 (62.0%) |  |
| 24-28 | 266 (33.3%) | 109 (31.9%) |  |
| ≥ 28 | 58 (7.26%) | 21 (6.14%) |  |
| CVC, No. (%) |  |  | 0.173 |
| No | 708 (88.6%) | 313 (91.5%) |  |
| Yes | 91 (11.4%) | 29 (8.48%) |  |
| ECOG, No. (%) |  |  | 0.856 |
| 0 point | 246 (30.8%) | 110 (32.2%) |  |
| 1 point | 423 (52.9%) | 175 (51.2%) |  |
| > 2 points | 130 (16.3%) | 57 (16.7%) |  |
| Histological type, No. (%) |  |  | 0.761 |
| Hodgkin | 75 (9.39%) | 34 (9.94%) |  |
| B cell | 583 (73.0%) | 256 (74.9%) |  |
| T cell | 72 (9.01%) | 25 (7.31%) |  |
| NK/T cell | 69 (8.64%) | 27 (7.89%) |  |
| Ann Arbor, No. (%) |  |  | 0.705 |
| Ⅰ-Ⅱ | 282 (35.3%) | 116 (33.9%) |  |
| Ⅲ-Ⅳ | 517 (64.7%) | 226 (66.1%) |  |
| WBC, No. (%) |  |  | 0.458 |
| < 11 ×10 ^9^/L | 688 (86.1%) | 288 (84.2%) |  |
| ≥ 11 ×10 ^9^/L | 111 (13.9%) | 54 (15.8%) |  |
| HB, No. (%) |  |  | 0.689 |
| ≥ 100 g/L | 642 (80.4%) | 279 (81.6%) |  |
| < 100 g/L | 157 (19.6%) | 63 (18.4%) |  |
| PLT, No. (%) |  |  | 0.803 |
| < 350 ×10 ^9^/L | 725 (90.7%) | 308 (90.1%) |  |
| ≥ 350 ×10 ^9^/L | 74 (9.26%) | 34 (9.94%) |  |
| D-dimer, No. (%) |  |  | 0.800 |
| ≤ 0.5 mg/L | 382 (47.8%) | 160 (46.8%) |  |
| > 0.5 mg/L | 417 (52.2%) | 182 (53.2%) |  |
| Chemotherapy cycles, No. (%) |  |  | 0.665 |
| 1-5 | 738 (92.4%) | 311 (90.9%) |  |
| 6-10 | 45 (5.63%) | 24 (7.02%) |  |
| ≥ 11 | 16 (2.00%) | 7 (2.05%) |  |
| Outcome, No. (%) |  |  | 1.000 |
| No VTE | 712 (89.1%) | 305 (89.2%) |  |
| VTE | 87 (10.9%) | 37 (10.8%) |  |

BMI: body mass index; CVC: central venous catheter; ECOG: Eastern Cooperative Oncology Group performance status; IQR: interquartile range; WBC: white blood cell count; HB: hemoglobin; PLT: Platelet count; VTE: venous thromboembolism

**Table S2.** Comparison of clinical demographics and clinicopathological characteristics between Non-VTE and VTE patients

| **Variables** | **non-VTE** | **VTE** | **p** |
| --- | --- | --- | --- |
| n | 1017 | 124 |  |
| VTE, No. (%) | 0 (0.0) | 124 (100.0) | <0.001 |
| Age, (median [IQR]) | 56.00 [47.00, 66.00] | 64.50 [54.00, 70.00] | <0.001 |
| Sex = female, No. (%) | 420 (41.3) | 43 (34.7) | 0.187 |
| BMI, No. (%) |  |  | 0.465 |
| < 24 | 618 (60.8) | 69 (55.6) |  |
| 24-28 | 331 (32.5) | 44 (35.5) |  |
| ≥ 28 | 68 (6.7) | 11 (8.9) |  |
| CVC = Yes, No. (%) | 64 (6.3) | 56 (45.2) | <0.001 |
| ECOG, No. (%) |  |  | 0.263 |
| 0 point | 316 (31.1) | 40 (32.3) |  |
| 1 point | 540 (53.1) | 58 (46.8) |  |
| > 2 points | 161 (15.8) | 26 (21.0) |  |
| Histological types, No. (%) |  |  | 0.029 |
| Hodgkin | 99 (9.7) | 10 (8.1) |  |
| B cell | 756 (74.3) | 83 (66.9) |  |
| T cell | 78 (7.7) | 19 (15.3) |  |
| NK/T cell | 84 (8.3) | 12 (9.7) |  |
| Ann Arbor = Ⅲ-Ⅳ, No. (%) | 656 (64.5) | 87 (70.2) | 0.251 |
| WBC≥ 11 ×10 9/L, No. (%) | 91 (8.9) | 74 (59.7) | <0.001 |
| HB < 100 g/L, No. (%) | 175 (17.2) | 45 (36.3) | <0.001 |
| PLT ≥ 350 ×10 9/L, No. (%) | 92 (9.0) | 16 (12.9) | 0.221 |
| D-dimer > 0.5 mg/L, No. (%) | 502 (49.4) | 97 (78.2) | <0.001 |
| Chemotherapy cycles, No. (%) |  |  | <0.001 |
| 1-5 | 954 (93.8) | 95 (76.6) |  |
| 6-10 | 45 (4.4) | 24 (19.4) |  |
| ≥ 11 | 18 (1.8) | 5 (4.0) |  |

**Table S3.** Clinical demographics and clinicopathologic characteristics of non-imputed data

| **Variables** | **Overall** |
| --- | --- |
| n | 1141 |
| Age (median [IQR]) | 56.00 [48.00, 66.00] |
| Sex, No. (%) |  |
| Female | 678 (59.4) |
| Male | 463 (40.6) |
| BMI, No. (%) |  |
| 24 | 682 (60.0) |
| 24-28 | 375 (33.0) |
| ≥ 28 | 79 (7.0) |
| CVC, No. (%) |  |
| No | 1021 (89.5) |
| Yes | 120 (10.5) |
| ECOG, No. (%) |  |
| 0 point | 351 (31.3) |
| 1 point | 587 (52.3) |
| > 2 points | 185 (16.5) |
| Histological type, No. (%) |  |
| Hodgkin | 109 (9.6) |
| B cell | 839 (73.5) |
| T cell | 97 (8.5) |
| NK/T cell | 96 (8.4) |
| Ann Arbor, No. (%) |  |
| Ⅰ-Ⅱ | 398 (35.0) |
| Ⅲ-Ⅳ | 739 (65.0) |
| WBC, No. (%) |  |
| < 11 ×10 ^9^/L | 976 (85.5) |
| ≥ 11 ×10 ^9^/L | 165 (14.5) |
| HB, No. (%) |  |
| ≥ 100 g/L | 920 (80.7) |
| < 100 g/L | 220 (19.3) |
| PLT, No. (%) |  |
| < 350 ×10 ^9^/L | 1033 (90.5) |
| ≥ 350 ×10 ^9^/L | 108 (9.5) |
| D-dimer, No. (%) |  |
| ≤ 0.5 mg/L | 542 (47.5) |
| > 0.5 mg/L | 599 (52.5) |
| Chemotherapy cycles, No. (%) |  |
| 1-5 | 1049 (91.9) |
| 6-10 | 69 (6.0) |
| ≥ 11 | 23 (2.0) |
| Outcomes, No. (%) |  |
| No VTE | 1017 (89.1) |
| VTE | 124 (10.9) |

BMI: body mass index; CVC: central venous catheter; ECOG: Eastern Cooperative Oncology Group performance status; IQR: interquartile range; WBC: white blood cell count; HB: hemoglobin; PLT: Platelet count; VTE: venous thromboembolism

**Table S4.** Performance evaluation of the six machine learning models in the training set and validation set

| **Training set** | Accuracy, 95%*CI* | Precision, 95%*CI* | Sensitivity, 95%*CI* | Specificity, 95%*CI* | F1 score, 95%*CI* | Brier score, 95%*CI* | AUC, 95%*CI* |
| --- | --- | --- | --- | --- | --- | --- | --- |
| Logistic regression | 0.76 [0.71, 0.80] | 0.84 [0.79, 0.88] | 0.65 [0.61, 0.68] | 0.87 [0.83, 0.90] | 0.73 [0.69, 0.77] | 0.4 [0.37, 0.42] | 0.88 [0.85, 0.91] |
| SVM | 0.81 [0.77, 0.85] | 0.81 [0.78, 0.84] | 0.83 [0.79, 0.86] | 0.79 [0.74, 0.82] | 0.82 [0.78, 0.85] | 0.19 [0.17, 0.21] | 0.90 [0.88, 0.92] |
| XGBoost | 0.90 [0.88, 0.91] | 0.91 [0.89, 0.94] | 0.88 [0.84, 0.90] | 0.92 [0.89, 0.94] | 0.90 [0.88, 0.92] | 0.13 [0.11, 0.14] | 0.96 [0.94, 0.97] |
| Random forest | 0.89 [0.81, 0.94] | 0.90 [0.86, 0.93] | 0.89 [0.86, 0.92] | 0.90 [0.87, 0.93] | 0.90 [0.86, 0.93] | 0.14 [0.12, 0.15] | 0.96 [0.93, 0.97] |
| Decision tree | 0.84 [0.80, 0.87] | 0.86 [0.83, 0.89] | 0.81 [0.77, 0.85] | 0.86 [0.81, 0.90] | 0.84 [0.80, 0.87] | 0.39 [0.36, 0.41] | 0.89 [0.86, 0.91] |
| BP-Network | 0.78 [0.75, 0.80] | 0.78 [0.73, 0.81] | 0.80 [0.76, 0.83] | 0.76 [0.72, 0.80] | 0.79 [0.72, 0.83] | 0.14 [0.12, 0.16] | 0.88 [0.84, 0.91] |
| **External validation set** | Accuracy, 95%*CI* | Precision, 95%*CI* | Sensitivity, 95%*CI* | Specificity, 95%*CI* | F1 score, 95%*CI* | Brier score, 95%*CI* | AUC, 95%*CI* |
| Logistic regression | 0.87 [0.85, 0.89] | 0.42 [0.39, 0.44] | 0.62 [0.58, 0.65] | 0.9 [0.86, 0.94] | 0.50 [0.46, 0.53] | 0.4 [0.37, 0.43] | 0.82 [0.79, 0.85] |
| SVM | 0.78 [0.74, 0.80] | 0.29 [0.26, 0.32] | 0.73 [0.70, 0.76] | 0.78 [0.74, 0.81] | 0.42 [0.39, 0.45] | 0.22 [0.20, 0.24] | 0.80 [0.77, 0.83] |
| XGBoost | 0.89 [0.87, 0.91] | 0.48 [0.44, 0.51] | 0.62 [0.58, 0.65] | 0.92 [0.89, 0.94] | 0.54 [0.51, 0.57] | 0.14 [0.13, 0.15] | 0.83 [0.81, 0.85] |
| Random forest | 0.86 [0.84, 0.89] | 0.40 [0.36, 0.43] | 0.68 [0.65, 0.72] | 0.88 [0.84, 0.92] | 0.51 [0.48, 0.54] | 0.17 [0.16, 0.18] | 0.82 [0.79, 0.84] |
| Decision tree | 0.74 [0.71, 0.76] | 0.24 [0.21, 0.27] | 0.68 [0.64, 0.72] | 0.74 [0.70, 0.78] | 0.36 [0.32, 0.40] | 0.18 [0.16, 0.19] | 0.80 [0.77, 0.83] |
| BP-network | 0.78 [0.75, 0.81] | 0.29 [0.26, 0.32] | 0.73 [0.70, 0.76] | 0.78 [0.73, 0.82] | 0.42 [0.38, 0.46] | 0.16 [0.14, 0.17] | 0.82 [0.78, 0.85] |

XGBoost: eXtreme Gradient Boosting; SVM: support vector machines; BP-network: backpropagation network; 95%*CI*: 95% Confidence Interval

**Table S5.** Normalized variable importance analysis for the six models by permutation importance analysis

| **variables** | XGBoost | Random forest | Decision tree | BP-network | SVM | Logistic regression |
| --- | --- | --- | --- | --- | --- | --- |
| WBC | 0.86 | 1.00 | 1.00 | 1.00 | 1.00 | 0.62 |
| CVC | 0.70 | 0.59 | 0.68 | 0.65 | 0.34 | 1.00 |
| D-dimer | 1.00 | 0.91 | 0.76 | 0.82 | 0.39 | 0.93 |
| Chemotherapy cycles | 0.38 | 0.21 | 0.25 | 0.45 | 0.32 | 0.23 |
| Age | 0.95 | 0.43 | 0.26 | 0.49 | 0.20 | 0.40 |
| ECOG | 0.57 | 0.47 | 0.00 | 0.53 | 0.22 | 0.01 |
| HB | 0.05 | 0.05 | 0.08 | 0.11 | 0.01 | 0.00 |
| Sex | 0.35 | 0.31 | 0.16 | 0.32 | 0.18 | 0.12 |
| Histological type | 0.32 | 0.27 | 0.11 | 0.14 | 0.06 | 0.20 |
| BMI | 0.22 | 0.20 | 0.00 | 0.23 | 0.14 | 0.00 |
| Ann Arbor | 0.11 | 0.06 | 0.00 | 0.00 | 0.09 | 0.00 |
| PLT | 0.00 | 0.00 | 0.00 | 0.15 | 0.00 | 0.02 |

XGBoost: eXtreme Gradient Boosting; SVM: support vector machines; BP-network: backpropagation network; BMI: body mass index; CVC: central venous catheter; ECOG: Eastern Cooperative Oncology Group performance status; IQR: interquartile range; WBC: white blood cell count; HB: hemoglobin; PLT: Platelet count

**Table S6**. Confusion matrix comparison of the VTE-EWS with KS in the external validation set

| Groups | **VTE-EWS** | |  | **KS** | |
| --- | --- | --- | --- | --- | --- |
|  | non-VTE | VTE |  | non-VTE | VTE |
| Low risk | 277 | 13 |  | 235 | 17 |
| High risk | 28 | 24 |  | 70 | 20 |

VTE: venous thromboembolism; KS: Khorana score; VTE-EWS: venous thromboembolism-early warning system

**Table S7.** Model performance comparation of the VTE-EWS with KS in the training set and external validation set

| **Training set** | Accuracy, 95%*CI* | Precision, 95%*CI* | Sensitivity, 95%*CI* | Specificity, 95%*CI* | AUC, 95%*CI* |
| --- | --- | --- | --- | --- | --- |
| **VTE-EWS** | 0.73 [0.70, 0.76] | 0.85 [0.80, 0.89] | 0.58 [0.53, 0.63] | 0.89 [0.86, 0.92] | 0.86 [0.84, 0.87] |
| **KS** | 0.61 [0.57, 0.64] | 0.68 [0.62, 0.73] | 0.44 [0.40, 0.49] | 0.78 [0.73, 0.82] | 0.66 [0.63, 0.70] |
| **External validation set** | Accuracy, 95%*CI* | Precision, 95%*CI* | Sensitivity, 95%*CI* | Specificity, 95%*CI* | AUC, 95%*CI* |
| **VTE-EWS** | 0.88 [0.84, 0.91] | 0.46 [0.33, 0.59] | 0.65 [0.49, 0.78] | 0.91 [0.87, 0.94] | 0.83 [0.75, 0.91] |
| **KS** | 0.75 [0.70, 0.79] | 0.22 [0.14, 0.31] | 0.54 [0.38, 0.69] | 0.77 [0.72, 0.81] | 0.69 [0.61, 0.78] |

AUC: area under curve; KS: Khorana score; VTE-EWS: venous thromboembolism-early warning system; 95%*CI*: 95% Confidence Interval

**Supplementary Figure**


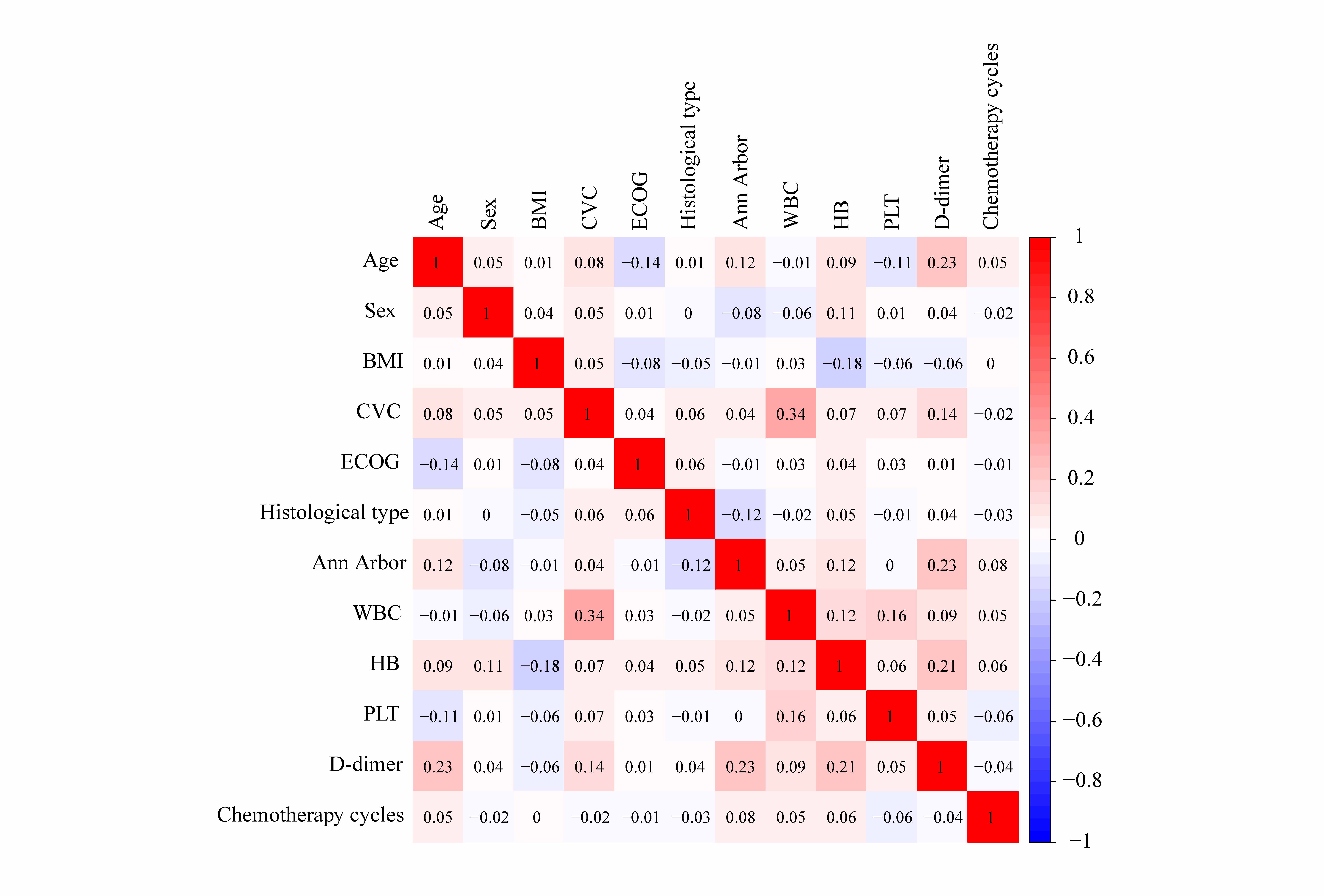
**Figure S1.** Heatmap analysis of variable correlations. BMI: body mass index; CVC: central venous catheter; ECOG: Eastern Cooperative Oncology Group performance status; WBC: white blood cell count; HB: hemoglobin; PLT: Platelet count


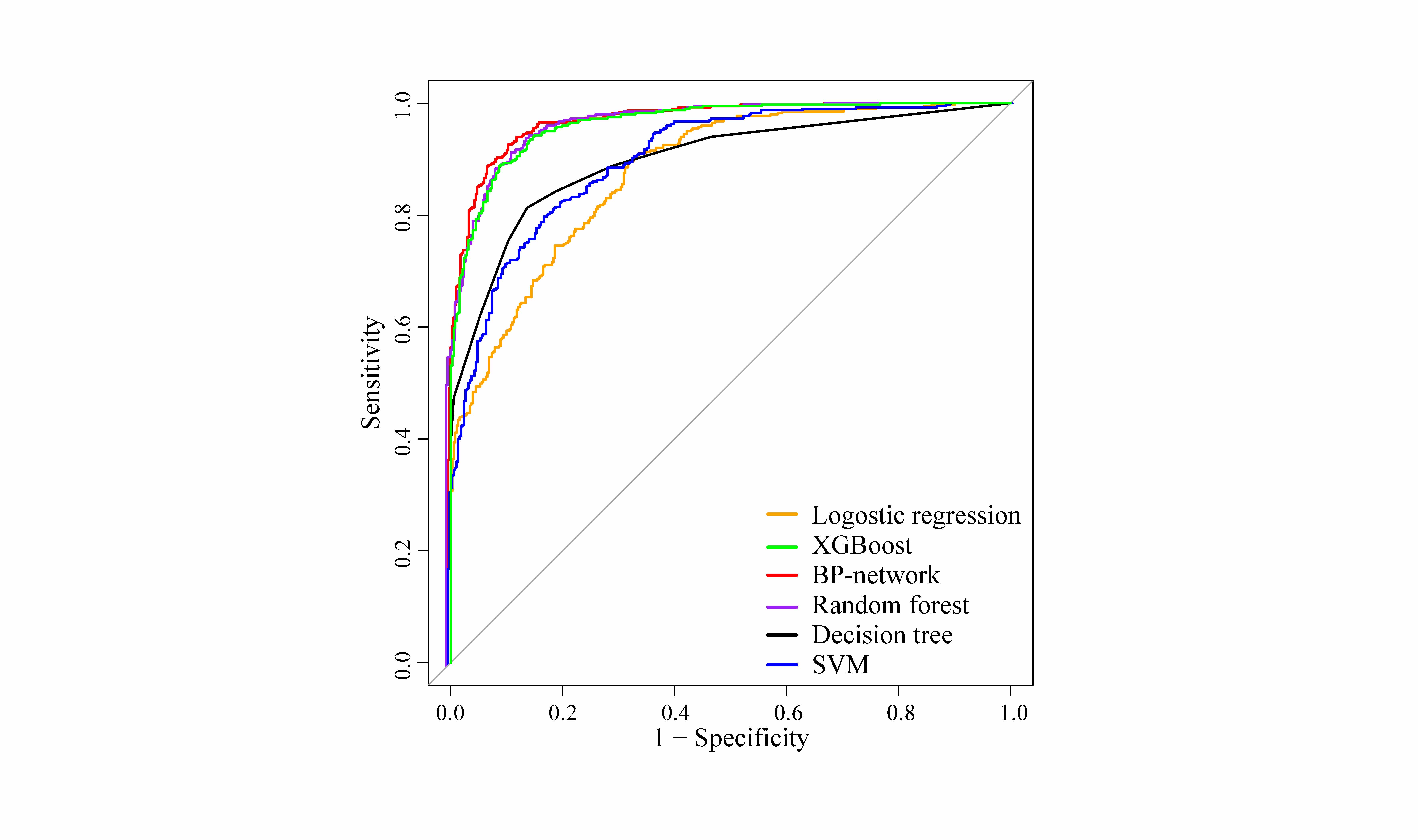


**Figure S2.** ROC curves for the six machine learning models in the training set. XGBoost: eXtreme Gradient Boosting; SVM: support vector machines; BP-network: backpropagation network; VTE: venous thromboembolism; ROC curve: receiver operator characteristic curve


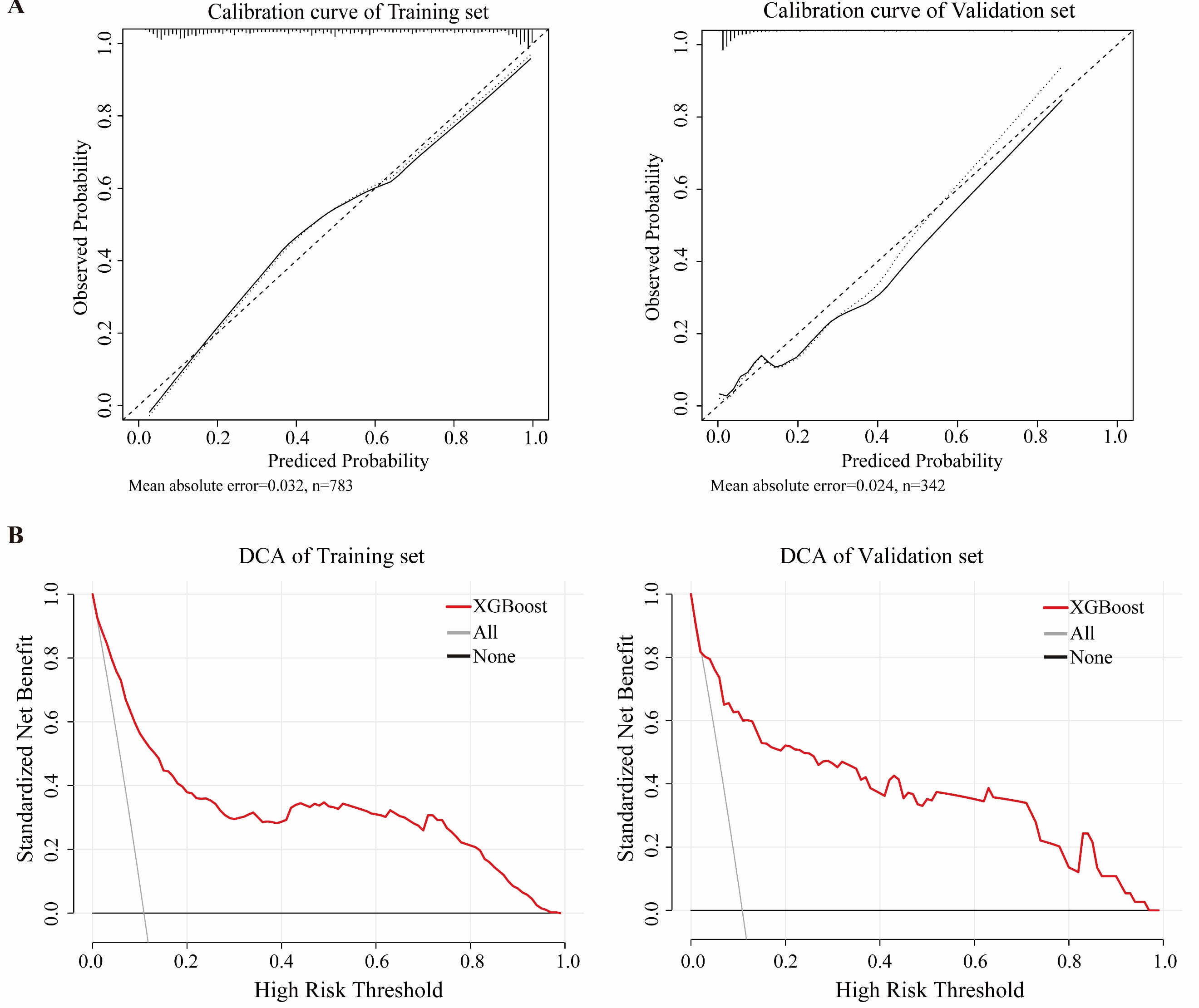


**Figure S3.** Model performance evaluation by Calibration curve and DCA. **(A)** Calibration curve of training set and validation set. **(B)** DCA of training set and validation set. DCA: decision curve analysis. XGBoost: eXtreme Gradient Boosting


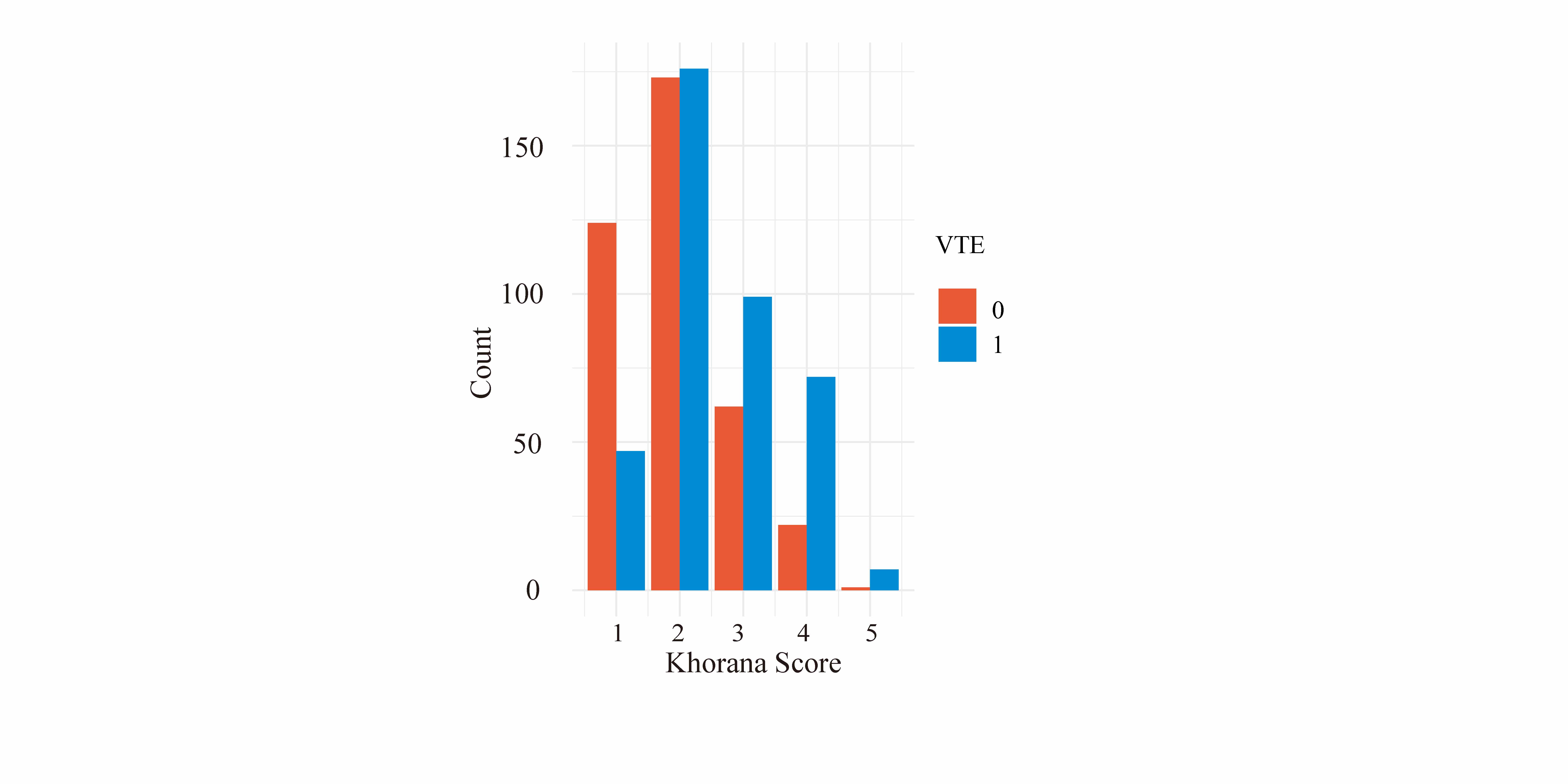


**Figure S4.** The number distribution of lymphoma patients undergoing chemotherapy under different KS scores. KS: Khorana score
